# SG-APSIC1203: Detection of carbapenemase genes in donor lungs at the point of care before transplantation reduces the risk of carbapenem-resistant Enterobacteriaceae–associated donor-derived infection in lung-transplant recipients

**DOI:** 10.1017/ash.2023.76

**Published:** 2023-03-16

**Authors:** Wenyong Zhou, Yi-Wei Tang

**Affiliations:** Shanghai Chest Hospital, Shanghai, China; Danaher Diagnostic Platform/Cepheid, Shanghai, China

## Abstract

**Objectives:** The number of lung transplants is increasing year by year in China and globally. With the widespread use of donation after brainstem death (DBD) donor lungs and donation after circulatory death (DCD) donor lungs, donor-derived infection (DDI) poses a major challenge in lung transplantation. Using donor lungs infected or colonized with carbapenem-resistant Enterobacteriaceae (CRE) may have serious implications in lung-transplant recipients. Currently, traditional microbial culture along with antimicrobial susceptibility testing cannot fully meet the need for rapid and accurate diagnosis of CRE infection in a donor before organ harvest. **Methods:** The Xpert Carba-R device (Cepheid, Sunnyvale, CA) was used to detect and differentiate *Klebsiella pneumoniae* carbapenemase (KPC), New Delhi metallo-β-lactamase (NDM), Verona integron-encoded metallo-β-lactamase (VIM), active-on-imipenem (IMP), and OXA-48 carbapenemase genotypes in bronchial lavage fluid from donor lungs before organ harvest. Positive detection of 1 or more of these genotypes indicated a potentially CRE-infected donor lung, and these organs were removed from the lung transplantation cohort. Donor lungs negative for all KPC, NDM, VIM, IMP, and OXA-48 genotypes determined by the Xpert Carba-R device were used for lung transplantation. The incidence of CRE-associated DDI and infection-related complications were compared in the Xpert Carba-R screening group and an historic control group. **Results:** In this study, 21 donor lungs were tested with the Xpert Carba-R device to detect and differentiate carbapenemase genotypes. Among them, 4 were positive for 1 or more carbapenemase genotypes and were discarded, and the remaining 17 donor lungs showing no carbapenemase gene presence were used for lung transplantation. No CRE-associated DDI occurred in these 17 lung-transplant recipients. **Conclusions:** Rapid and accurate detection of the carbapenemase gene in donor lungs at the point of care before transplantation using the Xpert Carba-R device reduced the risk of CRE-associated DDI in lung-transplant recipients.

